# Koller and the dawn of cancer cytogenetics

**DOI:** 10.1038/s41416-022-01996-z

**Published:** 2022-10-13

**Authors:** Chris J. Norbury

**Affiliations:** University of Oxford, Sir William Dunn School of Pathology, South Parks Road, Oxford, OX1 3RE UK

**Keywords:** Cancer, Genetics

## Abstract

Shortly before the DNA era began, PC Koller described lagging chromosomes and chromosome numerical abnormalities in human carcinomas. While present-day cancer geneticists would question some of Koller’s conclusions, this study ultimately contributed to the realisation that chromosomal instability is a widespread feature of solid tumours.

Seventy-five years ago the molecular basis of tumorigenesis was unknown and even the field of cytogenetics was in its infancy. Morgan and others had established by the early decades of the twentieth century that normal cellular function was associated with the duplication and accurate segregation of chromosomes, within which genetic determinants somehow resided. It was only with the refinement of cytological techniques in the 1950s, however, that it became clear that the normal human karyotype consisted of 23 pairs of chromosomes [1] and not 24, as was generally thought in 1947. In the article reviewed here, Pius Károly Koller, known as Peo Charles, working at the Chester Beatty Laboratories in London, documented a variety of mitotic abnormalities in human carcinomas [2]. Five years earlier Koller had developed a technique for resorcin blue staining of chromosomes in acetic acid-alcohol fixed biopsy specimens [3]. These preparations, once squashed (according to the protocol with the blunt end of a bone needle-holder!), could be visualised by direct photomicroscopy or by hand drawing, using a camera lucida device to view the specimen and drawing surface simultaneously.

Of the numerous observations made here using this technique, Koller’s description of the abundance of lagging (or ‘*sticky*’) chromosomes in mitotic carcinoma cells is perhaps the most significant and least contentious in the light of present-day understanding. Lagging chromosomes are now acknowledged to be a major underlying cause of mis-segregation and the consequent chromosomal instability characteristic of many solid tumours [4]. More recent studies have shed light on the causes of the ‘*stickiness*’ noted by Koller, including defects in sister chromatid cohesion, decatenation and cell cycle checkpoint responses, aspects of cell biology that were unknowable in the 1940s.

Yet in interpreting his findings Koller adheres rigidly to the assumption—shown by subsequent studies to be an over-generalisation—that ‘*loss of chromosomes or of chromosome segments leads to the death of the cell*’. For this reason, he hastily dismisses as ‘*erroneous*’ the earlier conclusions of Boveri, who these days is celebrated as having been the first to suggest that chromosome mis-segregation in a single progenitor cell might be fundamental to tumorigenesis [5]. The key point is that while mis-segregation may frequently lead to the death of one or both daughter cells, as Koller had previously suggested, it also provides a substrate for natural selection of any minority aneuploid progeny that have growth or survival advantages.

Some of the further claims made by Koller highlight technical limitations of his staining and imaging methodology, in particular the inability to distinguish between chromosomes or to count them accurately. For example, a mitotic figure from a rectal carcinoma is described as having only 16 chromosomes (‘*instead of 48*’), while a ‘*free giant tumour cell*’ aspirated from the abdomen has ‘*about 250 small chromosomes*’. Some of the images described may not represent intact chromosomes at all, but potentially apoptotic bodies, which had yet to be described, or simply artifacts of the fixation and staining procedure. Despite these issues, the data make a valuable contribution to the early characterisation of aneuploidy in carcinomas, now recognised as a general mechanism underlying intratumoral heterogeneity and the evolution of cancer phenotypes [6].

In clinging to the dogma that ‘*normal cellular activity stops when the nucleus does not contain the full chromosome complement*’, Koller feels obliged to explain the apparent ability of the aneuploid cells he describes to proliferate within the tumour. His chosen explanation revolves around the concept of cytoplasmic control, with malignant transformation involving some sort of critical cytoplasmic event that frees cells from the requirement to retain the normal karyotype. In support of this view, Koller notes that, in a rectal adenocarcinoma, there was ‘*synchronisation in the behaviour of adjacent cells*’, with 16 cells in one region undergoing simultaneous mitosis. This is taken as evidence of the aneuploid cells showing a ‘*great dependence*’ on each other and sharing a cytoplasmic driver of malignancy. The current consensus view of tumorigenesis through sequential acquisition of nuclear genomic changes has no place for rate-limiting heritable cytoplasmic changes, but Koller’s suggestion that an activator of mitosis (as opposed to transformation) might be shared between cells via the cytoplasm anticipates the identification in the 1980s of cyclin-dependent kinase 1 (CDK1) as the universal mitotic trigger [7]. It is unclear whether the synchronous mitoses seen by Koller reflected the presence of multinucleate cells or of intercellular connections somehow large enough to permit the free exchange of soluble proteins between adjacent cells.

Perhaps the most intriguing claim made by Koller is that mitotic abnormalities are more frequent in poorly vascularised regions of carcinomas than in rapidly proliferating, peripheral regions. He cites inadequate ‘*food supply*’ and the possible involvement of ‘*toxic breakdown products*’ as potential underlying causes of chromosome mis-segregation. While the relationship between cell nutrition and mitotic fidelity remains rather poorly understood to this day, especially in the context of primary human tumours, recent literature has highlighted mechanisms by which hypoxia and/or oxidative base damage may generate lagging chromosomes [8, 9], providing support for Koller’s hypothesis.

Koller’s chromosomal account of cancer biology is completely separable from the current DNA-centric view. Only three years earlier Avery, MacLeod and McCarty had demonstrated the of role of DNA in bacterial transformation [10], though the generality of this finding in relation to inheritance was not universally acknowledged in 1947. Koller instead regards ‘*nucleic acid*’ as being important for chromosome organisation and condensation, and even suggests that chromosome stickiness might be due to an ‘*excess of nucleic acid charge*’, a concept wholly at odds with current understanding of nucleic acid chemistry and chromatin structure.

Progress in cancer research can often feel painfully slow, but this article is a useful reminder of just how far the field has advanced in the past 75 years. At the same time, it underscores the inherent advantages of studies based on direct observation of human tumours and hints at lines of investigation that may even now offer further insight into the fidelity of chromosome segregation in different tumour microenvironments.
